# Identification and validation of key genes in gastric cancer: insights from *in silico* analysis, clinical samples, and functional assays

**DOI:** 10.18632/aging.205965

**Published:** 2024-06-23

**Authors:** Xiaofeng Pei, Yuanling Luo, Huanwen Zeng, Muhammad Jamil, Xiaodong Liu, Bo Jiang

**Affiliations:** 1 Department of Oncology, The Fifth Affiliated Hospital of Sun Yat-sen University, Zhuhai 519000, China; 2 PARC Arid Zone Research Center, Dera Ismail Khan 29050, Pakistan; 3 Department of Pharmacy, The 922 Hospital of Joint Logistics Support Force, PLA, Hengyang 421002, China; 4 Department of Emergency, The Fifth Affiliated Hospital of Sun Yat-sen University, Zhuhai 519000, China

**Keywords:** gastric cancer, biomarker, expression analysis, differentially expressed genes

## Abstract

Introduction: The underlying mechanisms of gastric cancer (GC) remain unknown. Therefore, in this study, we employed a comprehensive approach, combining computational and experimental methods, to identify potential key genes and unveil the underlying pathogenesis and prognosis of GC.

Methods: Gene expression profiles from GEO databases (GSE118916, GSE79973, and GSE29272) were analyzed to identify DEGs between GC and normal tissues. A PPI network was constructed using STRING and Cytoscape, followed by hub gene identification with CytoHubba. Investigations included expression and promoter methylation analysis, survival modeling, mutational and miRNA analysis, gene enrichment, drug prediction, and *in vitro* assays for cellular behaviors.

Results: A total of 83 DEGs were identified in the three datasets, comprising 41 up-regulated genes and 42 down-regulated genes. Utilizing the degree and MCC methods, we identified four hub genes that were hypomethylated and up-regulated: COL1A1, COL1A2, COL3A1, and FN1. Subsequent validation of their expression and promoter methylation on clinical GC samples through targeted bisulfite sequencing and RT-qPCR analysis further confirmed the hypomethylation and overexpression of these genes in local GC patients. Furthermore, it was observed that these hub genes regulate tumor proliferation and metastasis in *in vivo* and exhibited mutations in GC patients.

Conclusion: We found four potential diagnostic and prognostic biomarkers, including COL1A1, COL1A2, COL3A1, and FN1 that may be involved in the occurrence and progression of GC.

## INTRODUCTION

Gastric cancer (GC) stands as the sixth most frequently diagnosed cancer globally, with the second-highest mortality rate among malignant tumors [1]. While the 5-year overall survival rate for early-stage GC patients can reach 95% [2], it remains around 50% for those in the advanced stage, even with comprehensive treatment approaches involving surgery [2, 3]. The low survival rate of GC is primarily attributed to tumor recurrence and metastasis [4]. As a result, it becomes crucial to delve into the potential molecular mechanisms that drive the malignant biological behavior of GC cells. Moreover, the discovery of efficient early diagnostic techniques and dependable molecular markers for recurrence monitoring and prognosis evaluation holds significant importance. Despite notable progress in comprehending the molecular intricacies of GC and the emergence of targeted therapeutic options, the effectiveness of existing targeted therapies remains limited for certain patients [5, 6]. Therefore, further research aimed at uncovering novel and more effective targeted approaches is essential to improve patient outcomes and overcome these challenges.

In recent years, the utilization of microarray and RNA-sequencing technology has emerged as a powerful and efficient tool in the quest for promising biomarkers to aid in cancer diagnosis, treatment, and prognosis [7, 8]. These technologies have led to the accumulation of a vast amount of data, accessible through public database platforms like Gene Expression Omnibus (GEO) [9] and The Cancer Genome Atlas (TCGA) [10, 11]. By leveraging the wealth of information in GEO and TCGA, scientists can uncover novel molecular signatures and candidate biomarkers that may have diagnostic, prognostic, or therapeutic implications in the fight against cancer [12]. The integration of GEO data with other experimental approaches enables a deeper understanding of cancer biology and aids in the early detection and treatment of cancer. By performing experimental validation, researchers can verify the expression patterns, molecular interactions, and functional roles of the identified biomarkers, ultimately strengthening the confidence in their potential clinical utility.

Various investigations have been undertaken to analyze the abnormal gene expression patterns associated with GC. Despite advanced research, these studies yielded inconsistent results [13–16]. Therefore, to address the challenges posed by diverse technological platforms and small sample sizes, integrated bioinformatics approaches have been embraced in cancer studies, yielding a wealth of valuable biological insights. In pursuit of gaining profound insights into the influence of Differentially Expressed Genes (DEGs) on the molecular pathogenesis of GC, our study aimed to explore novel signature genes associated with GC. For this purpose, we adopted a multi-level validation approach to rigorously examine and confirm the relevance and significance of these signature genes in the context of GC.

## MATERIALS AND METHODS

### Methodology

**Figure 1:**
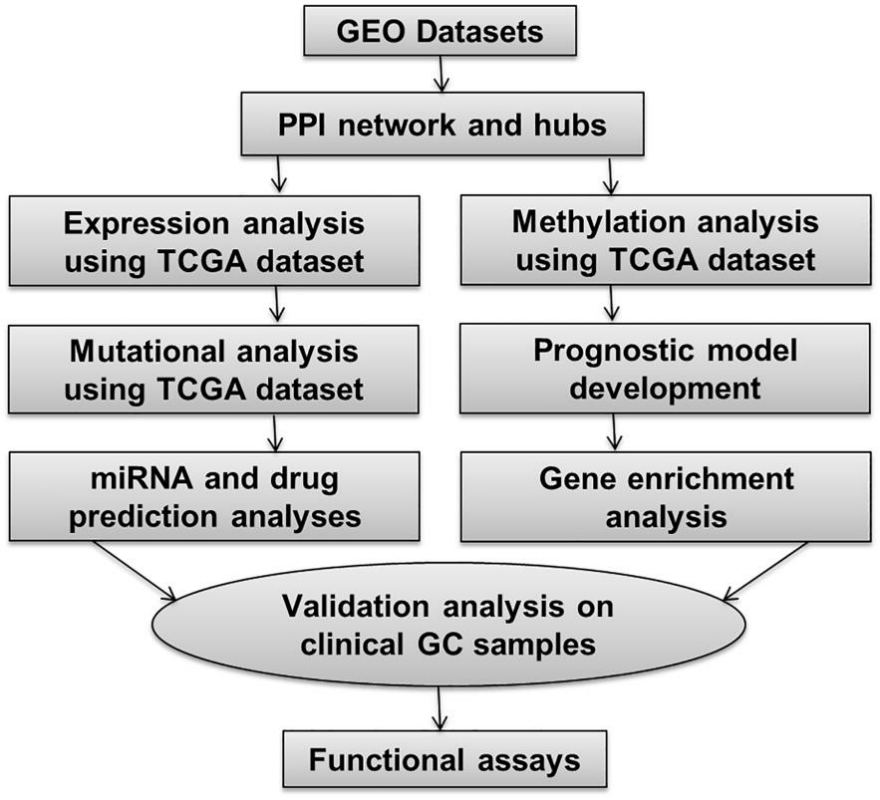
This figure illustrates the overall methodology utilized in the present study.

The overall methodology employed in this study is depicted in Figure 1.

### Collection of clinical specimens

We acquired paired fresh cancer tissue specimens along with control samples from 39 patients who underwent surgical resection of GC at the District Headquarter Hospital (DHQ), Teaching Hospital, Dera Ismail Khan, Khyber Pakhtunkhwa (KPK) between 2019 and 2023. None of the patients had received any chemotherapy or radiation therapy prior to the surgery. The collected tissue samples were promptly frozen in liquid nitrogen and stored at −80°C until DNA and RNA isolation. The study received ethical approval in accordance with the Helsinki Declaration, and informed written consent was obtained from all participants.

### Microarray data acquisition, DEGs, and hub genes identification

We retrieved three datasets of microarray, namely GSE118916, GSE79973, and GSE29272, from the GEO database at http://www.ncbi.nlm.nih.gov/geo/. The selection criteria for appropriate GC datasets were as follows: studies involving pharmacological manipulation, interfering molecules like miRNAs, siRNAs, or gene therapies, knockdown cultures, or artificially induced mutations were excluded. Only studies with a minimum of ten control and experimental samples, exclusively conducted in Homo sapiens, and providing clear descriptions of protocols or samples were chosen. Additionally, datasets with raw data availability, excluding those with treated data only, and studies conducted on platforms belonging to Affymetrix, Illumina, or Agilent manufacturers were included. Samples from metastasized tissues were also excluded. A total of 16 microarray datasets were reviewed, and GSE118916, GSE79973, and GSE29272 were selected based on sample size adequacy for further analysis.

For our study, we specifically selected paired GC tissues and their corresponding adjacent tissues. In cases where multiple probes were associated with a particular gene, we calculated the average expression level to represent its final expression. The initial microarray data from each series underwent processing using the R software package (version 3.6.1; http://www.r-project.org/). Following the transformation to a log2 scale, we set the cutoff criteria for identifying DEGs as |Log2 fold change (FC)| > 1 and adjusted *P* < 0.01. To visualize the common DEGs across the three datasets, we generated a Venn diagram utilizing Venny (version 2.1; https://bioinfogp.cnb.csic.es/tools/venny/index.html). Subsequently, we selected these overlapping DEGs for further investigation of hub genes.

In order to identify hub genes from the overlapping DEGs, we first constructed a protein-protein interaction (PPI) network using the STRING database (https://string-db.org/) [17]. During PPI construction all the active interactions sources were utilized including textmining, experiments, database, co-expression, neighborhood, genefusion, and co-occurrence with 1^st^ shell. The minimum required interaction score was set to 0.4 (medium confidence) and the protein nodes having no interaction with other proteins were removed from the network. This allowed us to explore the interactions and associations between the identified genes. Next, we employed the CytoHubba application within the Cytoscape software to analyze the PPI network and pinpoint the hub genes based on the degree method [18].

### TCGA-datasets-based expression validation analysis

In this study, we harnessed the power of three crucial databases, including UALCAN (https://ualcan.path.uab.edu/cgi-bin/ualcan-res.pl) [19], OncoDB (https://oncodb.org/) [20], and GEPIA (http://gepia.cancer-pku.cn/) [21] to validate the expression of hub genes on the GC TCGA expression datasets. UALCAN offers a user-friendly interface, providing researchers with valuable insights into gene expression patterns and conducting in-depth analyses using TCGA data. OncoDB serves as a comprehensive repository of oncogenomic information, shedding light on cancer-related gene expression and molecular alterations. GEPIA, on the other hand, is a web-based tool enabling researchers to explore gene expression data and conduct interactive analyses across diverse cancer types.

### Proteomic expression analysis

In this study, the UALCAN database was used to conduct proteomic expression analysis of the hub genes across GC and normal tissues. By utilizing this database, we gained essential information on the protein expression patterns of these hub genes, enhancing our understanding of their potential roles in GC and normal tissue biology.

### Promoter methylation analysis

In our research, we utilized the promoter methylation features available in the UALCAN [19] and OncoDB [20] databases. These databases provide essential information on the epigenetic modifications of genes, particularly focusing on promoter methylation, which can regulate gene expression. Leveraging the data from these resources, we conducted a comprehensive promoter methylation analysis of the hub genes identified across GC and normal tissue samples. By examining the methylation patterns of these hub genes, we aimed to gain valuable insights into potential regulatory mechanisms that could influence gene expression in the context of GC.

### Mutational and co-expressed gene analyses

In the current study, we utilized the mutational analysis feature of the cBioPortal database (https://www.cbioportal.org/) [22], a powerful resource for exploring genomic alterations in various cancers. cBioPortal provides comprehensive genomic and clinical data from numerous cancer studies, enabling researchers to assess the mutation landscape of specific genes of interest. We conducted an extensive mutational analysis of the hub genes across GC samples using cBioPortal with default settings. By examining the mutational status of these hub genes, we aimed to uncover potential genetic alterations that could influence their functions and contribute to the development and progression of GC. In addition to this, we also used this database with default settings to identify mutually co-expressed genes with hub genes in GC.

### Survival analysis and the construction of a prognostic model

In this study, we employed two important methodologies to explore the prognostic implications of the hub genes. Firstly, we utilized the GEPIA [21], a powerful online tool for conducting survival analysis, to assess the association between the expression levels of the hub genes and patient outcomes. GEPIA allowed us to investigate the impact of these genes on overall survival and disease-free survival in specific cancer types. Secondly, to construct a robust prognostic model, we implemented the Cox regression method [23] via R. This approach enabled us to develop a predictive model that could accurately stratify patients based on their risk of adverse clinical outcomes.

### Enrichment and miRNA prediction analyses

In our study, we utilized three important databases, namely DAVID, [24] miRDB, and ENCORI [25], to gain further insights into the functional implications of the hub genes identified. DAVID (Database for Annotation, Visualization, and Integrated Discovery) offers a comprehensive platform for gene functional annotation and enrichment analysis. We performed Gene Ontology (GO) and Kyoto Encyclopedia of Genes and Genomes (KEGG) pathway analysis of the hub genes using the DAVID database. We utilized miRDB and ENCORI database to conduct miRNA prediction analysis of the hub genes.

### Genomic DNA and RNA isolation

Total cell DNA from tissue samples was extracted using an organic method [26], while total RNA was extracted using TRIZol method [27]. We employed the NanoDrop 2000 Spectrophotometer (Thermo Fisher Scientific, Waltham, MA, USA) to assess the concentration and purity of the extracted DNA and RNA, ensuring that the A260/A280 ratio fell within the range of 1.8 to 2.0.

### Library preparation for targeted bisulfite sequencing analysis

In brief, total DNA (1 μg) was fragmented into approximately 200–300 bp fragments using a Covarias sonication system (Covarias, Woburn, MA, USA). Following purification, the DNA fragments underwent repair and phosphorylation of blunt ends using a mixture of T4 DNA polymerase, Klenow Fragment, and T4 polynucleotide kinase. The repaired fragments were then 3′ adenylated using Klenow Fragment (3′–5′ exo-) and ligated with adapters containing 5′-methylcytosine instead of 5′-cytosine and index sequences using T4 DNA Ligase. The constructed libraries were quantified using a Qubit fluorometer with the Quant-iT dsDNA HS Assay Kit (Invitrogen, Carlsbad, CA, USA) and sent to Beijing Genomic Institute (BGI), China for targeted bisulfite sequencing. Following sequencing, the methylation data was normalized into beta values.

### Real time quantitative PCR (RT-qPCR)

The RNA extracted was transcribed into cDNA using the Prime-Script RT reagent kit (TaKaRa, Dalian, China). RT-qPCR analysis was performed on an ABI 7500 Real-Time PCR System (Applied Biosystems, USA) using the SYBR Premix Ex Taq^™^ II kit (TaKaRa). The expression levels were normalized to β-actin. All experiments were independently conducted in triplicate. The 2^(−ΔΔCt)^ method was employed to assess the relative expression of each hub gene [28]. This method quantifies gene expression changes by comparing the cycle threshold (Ct) values of a target gene between control and experimental groups. It normalizes to reference genes, yielding a fold change value (2^(−ΔΔCt)^), indicating whether the gene is up-regulated (>1), down-regulated (<1), or unchanged (=1).

### Receiver operating characteristic (ROC) curve generation

Based on the RT-qPCR and targeted bisulfite-seq expression and methylation data, ROC curves of identified DEGs expression were generated using the SRPLOT web source (https://bioinformatics.com.cn/srplot).

### Drug prediction analysis

In our study, we harnessed the drug prediction feature of the DrugBank (http://www.drugbank.ca) database [29], a comprehensive resource that provides valuable information on drug-target interactions and drug-related data. Leveraging this feature, we aimed to identify potential drugs that could target the hub genes identified in our study. By exploring the vast database of drug-target interactions, we sought to uncover drugs that may have regulatory effects on the expression of the hub genes.

### Cell culture and transfection

AGS cell line (obtained from the American Type Culture Collection, ATCC, Manassas, VA, USA) were maintained in a 37°C incubator with 5% CO_2_ in Dulbecco’s Modified Eagle Medium (DMEM) from Hyclone (Logan, UT, USA) supplemented with 10% fetal bovine serum (FBS) obtained from Gibco (Waltham, MA, USA). Following this, we carried out gene knockdown experiments targeting COL1A1, COL1A2, COL3A1, and FN1 genes. These knockdowns were achieved by transfecting the cell with two siRNA constructs specific to each gene-namely, si-COL1A1, si-COL1A2, si-COL3A1, and si-FN1. The transfection was facilitated using Lipofectamine 3000 from Invitrogen (Waltham, MA, USA). The cells were subsequently cultured for an additional 48 hours following transfection.

Following are the sequences of the utilized siRNAs:

si-COL1A1-1: 5′-TTGGTGTTGTGCGATGACGTG-3′; si-COL1A1-2: 5′-GTACGTCCGGTTGTATGTA-3′; si-COL1A2-1: 5′-GGACCCGTTGGCAAAGATG-3′; si-COL1A2-2: 5′-CACCAGGAGGACCAGGAG-3′; si-COL1A3-1: 5′-CUAUGCGGAUAGAGAUGUCTT-3′; si-COL1A3-2: 5′-GAGGAAACAGAGGTGAAAGA GG-3′; si-FN1-1 sense: 5′-CCAUUUCACCUU CAGACAATT-3′; si-FN1-1 anti-sense: 5′-UUGU CUGGGUGAAAUGGTT-3′; si-FN2-1 sense: 5′-GCAAGCAGCAACAAUUUTT-3′; si-FN2-1 anti-sense: 5′-AAAUUGGCUUGCUGAUUGCTT-3′.

### RNA extraction and RT-qPCR

Total RNA from the cell lines was extracted using TRIZol method [30] and RT-qPCR analysis was performed according to the instructions as discussed above.

### Cell counting kit-8 (CCK-8) assays

After the transfection process, AGS cells were plated in 96-well plates at a concentration of 1 × 10^5^ cells/mL and allowed to proliferate for 48 hours. To assess cell viability, we employed a CCK-8 kit (provided by Meilunbio, China), following the manufacturer’s instructions. Absorbance measurements at 450 nm were taken using a Bio-Rad model 550 microplate reader.

### Colony-forming assays

The cells were distributed into 6-well plates, with each well receiving 500 cells, and were then cultured for 48 hours. Subsequently, the cells were exposed to the correct doses of ATO (2 μM for AGS cells). Following one-week incubation, the cells were immobilized using 4% paraformaldehyde sourced from Thermo Fisher Scientific (Waltham, MA, USA). Afterward, they were subjected to staining with 2% crystal violet from Thermo Fisher Scientific (USA). Colonies that were clearly visible and consisted of at least 50 cells per clone were enumerated under a microscope.

### Statistics analysis

DEGs were identified using a *t*-test [31]. While for GO and KEGG enrichment analysis, we used Fisher’s Exact test for computing difference [32]. Correlational analyses were carried out using the Pearson method. For comparisons, a student *t*-test was adopted in the current study. All the analyses were carried out in R version 3.6.3 software.

### Availability of data and materials

The data supporting the findings of the article are available in the GEO database at https://www.ncbi.nlm.nih.gov/geo/.

## RESULTS

### Microarray data acquisition, DEGs, and hub genes identification

**Figure 2:**
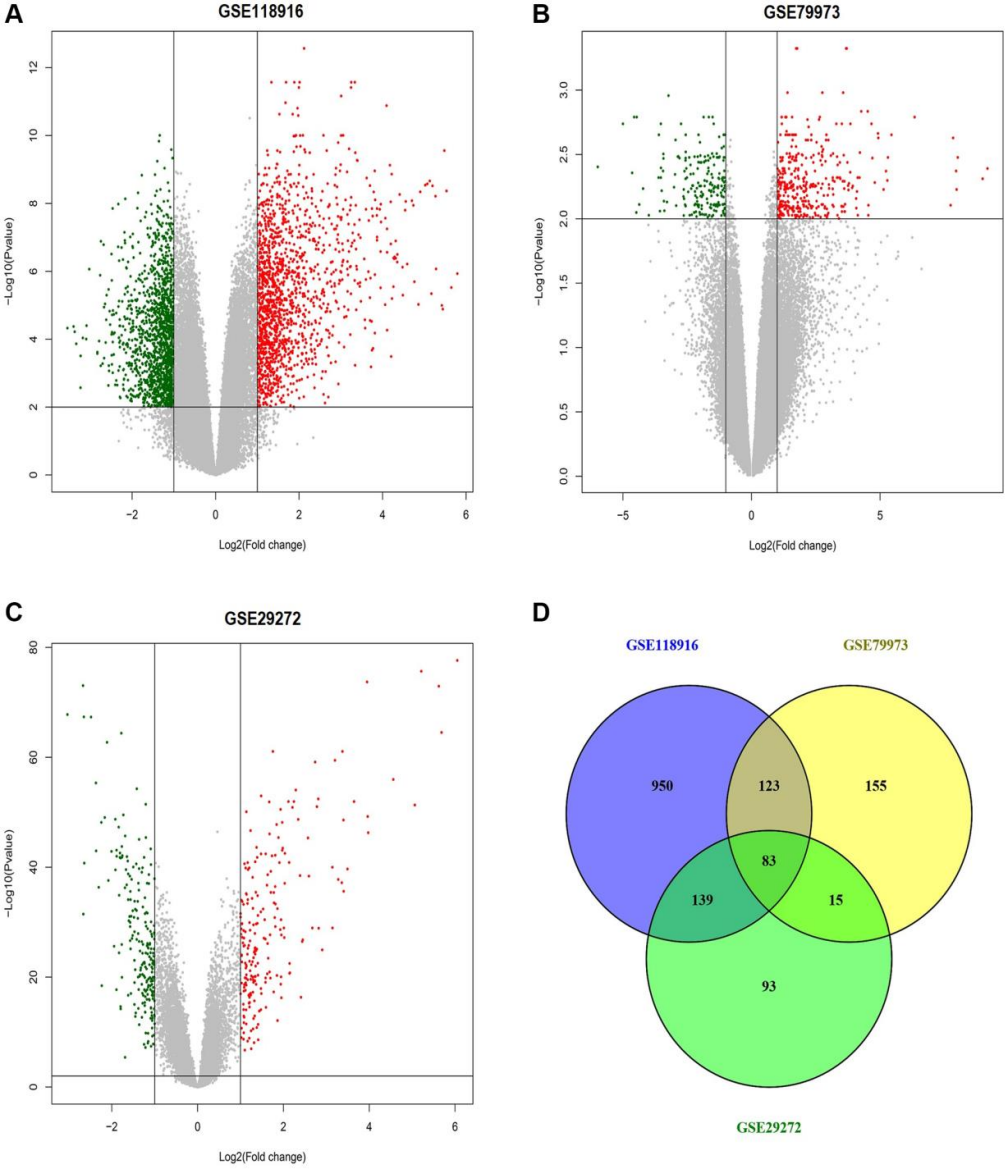
**This figure depicts the process of identifying differentially expressed genes (DEGs) across the GSE118916, GSE79973, and GSE29272 datasets related to gastric cancer (GC).** (**A**) Volcano plot of differentially expressed genes (DEGs) in the GSE118916 dataset. (**B**) Volcano plot of DEGs in the GSE79973 dataset. (**C**) Volcano plot of DEGs in the GSE29272 dataset. (**D**) Venn diagram showing the overlap of DEGs among the three datasets (GSE118916, GSE79973, and GSE29272). Red dots represent up-regulated genes, and green dots represent down-regulated genes. The numbers in Venn diagram represent the count of unique and overlapping genes among the datasets. *P*-value < 0.05.

We obtained three gene expression profiles (GSE118916, GSE79973, and GSE29272) from the GEO database, comprising a total of 318 samples, including 159 GC and 159 matched adjacent control tissues. From GSE118916, a total of 1295 DEGs were identified, consisting of 651 up-regulated and 644 down-regulated genes. In GSE79973, a total of 376 DEGs were screened, including 132 up-regulated and 244 down-regulated genes. Similarly, GSE29272 yielded 330 DEGs, comprising a total of 165 up-regulated and 165 down-regulated genes. Volcano plots depicting the DEGs in each dataset were illustrated in Figure 2A–2C. Among these datasets, 83 genes (41 up-regulated and 43 down-regulated) were common, and were chosen for further analysis (Figure 2D).

**Figure 3:**
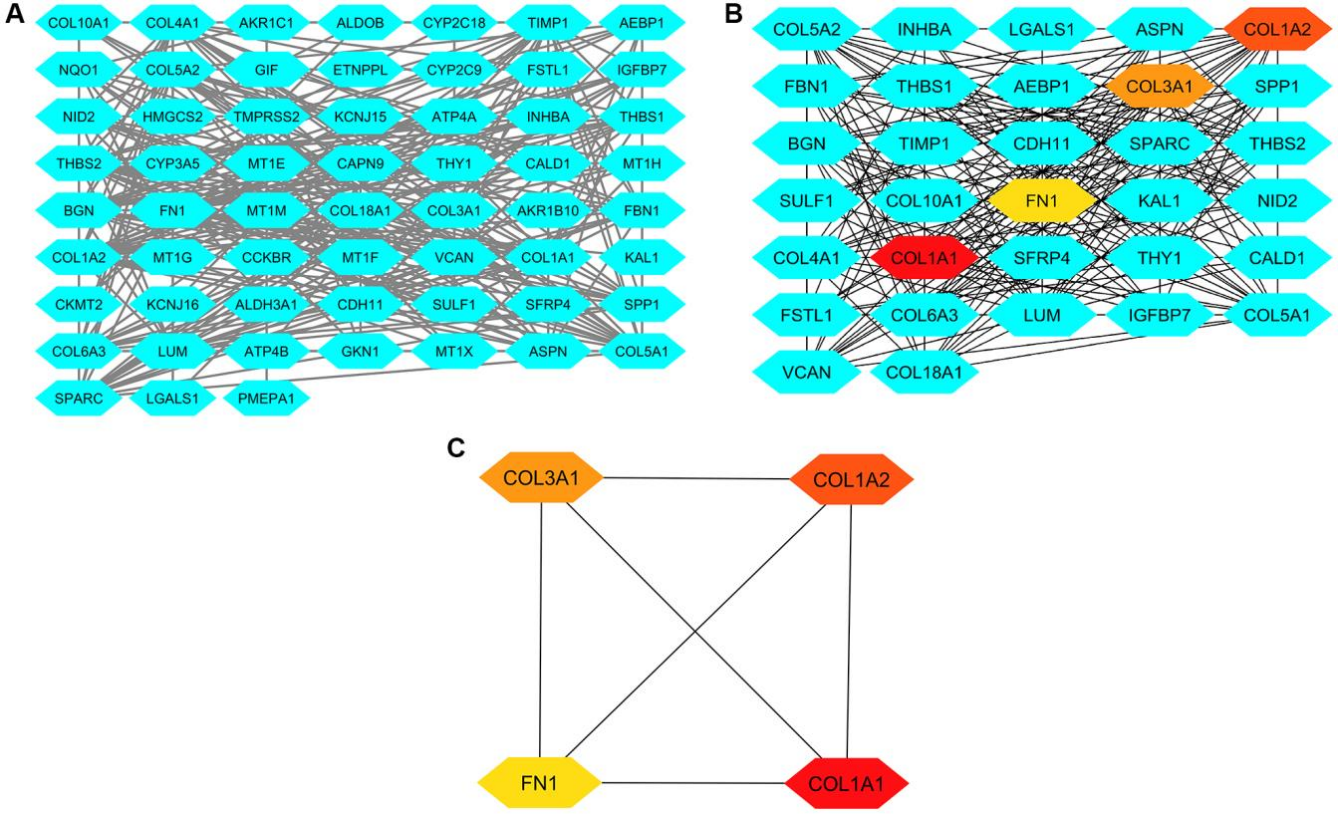
**This figure illustrates the process of constructing protein-protein interaction (PPI) networks, analyzing them, and identifying hub genes.** (**A**) Panel A presents the PPI network formed by the 83 common DEGs from GSE118916, GSE79973, and GSE29272. (**B**) Panel B displays the PPI network of these DEGs highlighting hub genes identified through degree and MCC methods. (**C**) Panel C showcases a refined PPI network focusing solely on the four identified hub genes.

To investigate the interactions between the 83 DEGs, we utilized the STRING database to construct a PPI network. The resulting PPI network was generated using Cytoscape (Figure 3A) and comprised 83 nodes with 253 interactions (Figure 3A). Subsequently, the PPI network was analyzed using the cytoHubba application in Cytoscape to identify hub genes (Figure 3B). This analysis involved two algorithms, degree and MCC, provided by cytoHubba. Based on these two algorithms, up-regulated COL1A1 (Collagen, type I, alpha 1), COL1A2 (Collagen, type I, alpha 2), COL3A1 (Collagen, type III, alpha 1), and FN1 (Fibronectin) in GC samples were identified as the hub genes (Figure 3C). These genes exhibited significant connectivity within the network, suggesting their potential importance in the regulatory network of the DEGs.

### Expression validation based on TCGA datasets

**Figure 4:**
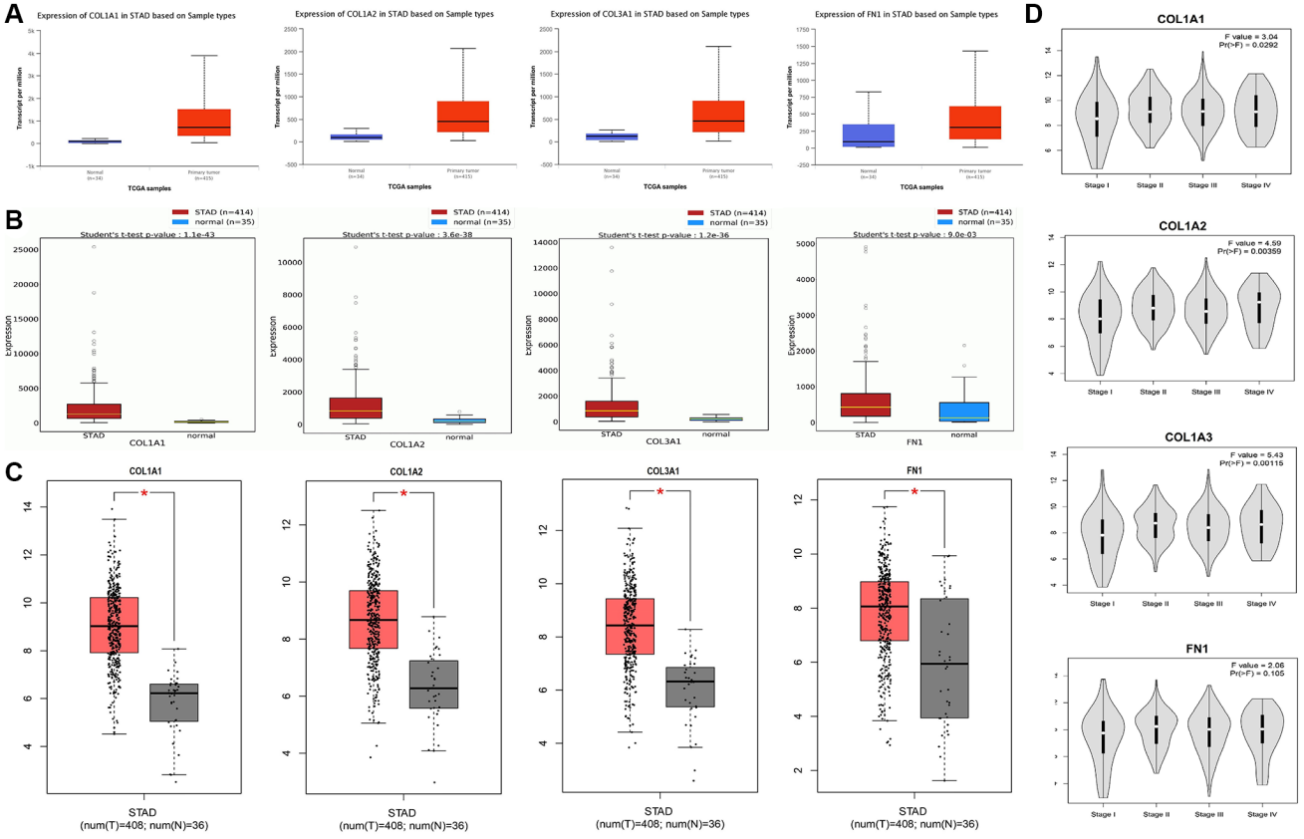
**mRNA expression analysis of COL1A1, COL1A2, COL3A1, and FN1 using additional TCGA datasets of gastric cancer (GC).** (**A**) Expression analysis of COL1A1, COL1A2, COL3A1, and FN1 in GC and normal samples via UALCAN database. (**B**) Expression analysis of COL1A1, COL1A2, COL3A1, and FN1 in GC and normal samples via OncoDB database. (**C**) Expression analysis of COL1A1, COL1A2, COL3A1, and FN1 in GC and normal samples via GEO GEPIA. (**D**) Expression analysis of COL1A1, COL1A2, COL3A1, and FN1 in GC samples belonging to different cancer stages. *P*-value < 0.05.

To confirm the mRNA expression levels of COL1A1, COL1A2, COL3A1, and FN1 in GC samples compared to controls from the TCGA database, we utilized UALCAN, OncoDB, and GEPIA for data integration and visualization. These hub genes (COL1A1, COL1A2, COL3A1, and FN1) exhibited significant overexpression (*p* < 0.05) in GC samples relative to controls (Figure 4A–4C), which was consistent with the findings from the GEO datasets. Furthermore, the expression of COL1A1, COL1A2, COL3A1, and FN1 varied across different stages of GC (Figure 4D). These results provide additional evidence supporting the up-regulation of these hub genes in GC.

### Proteomic expression analysis of COL1A1, COL1A2, COL3A1, and FN1

**Figure 5:**
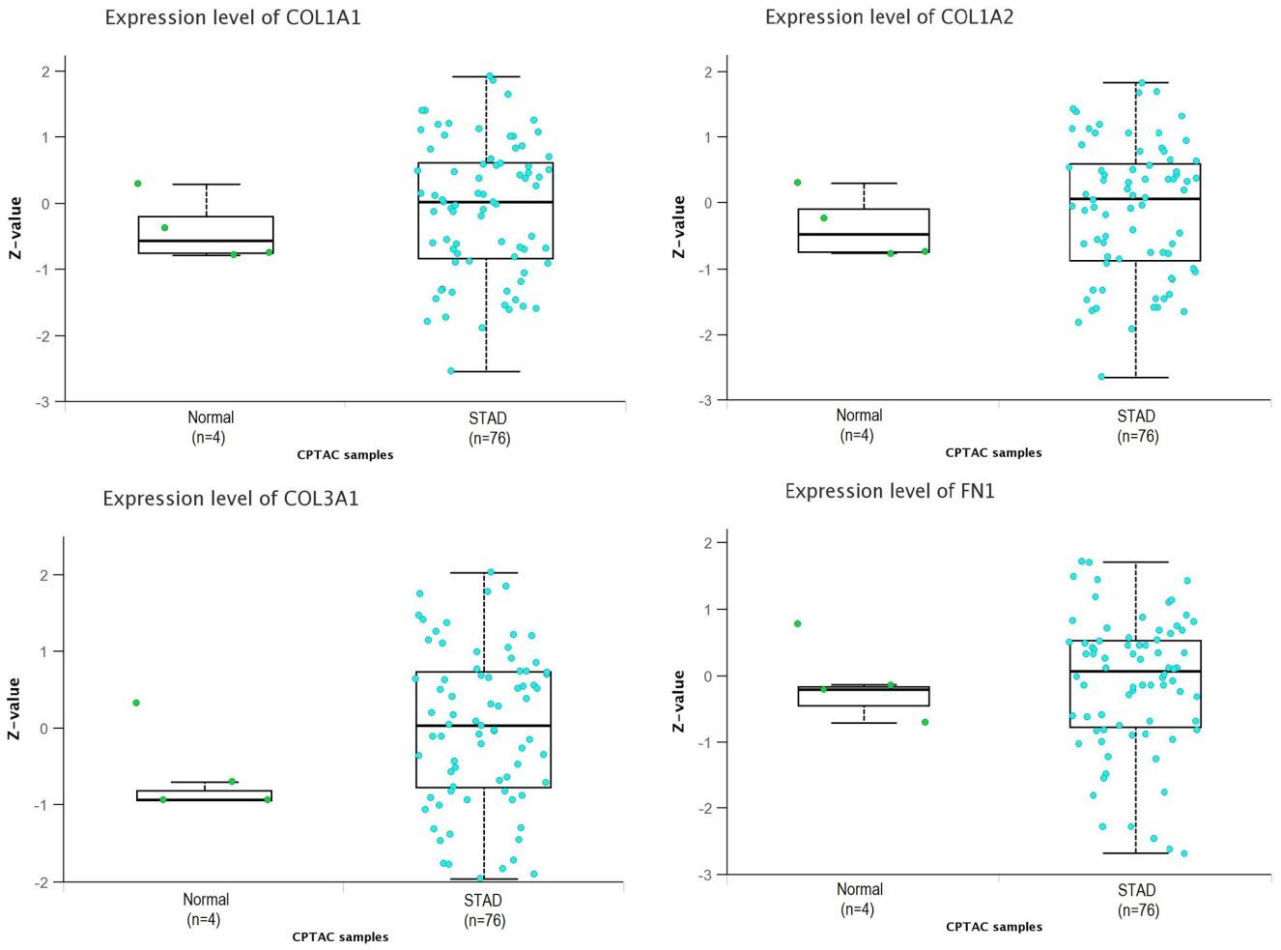
**Proteomic expression analysis of COL1A1, COL1A2, COL3A1, and FN1 using additional database.** This figure presents the proteomic expression analysis of COL1A1, COL1A2, COL3A1, and FN1 in gastric cancer (GC) and normal samples via UALCAN database. *P*-value < 0.05.

In our study, we conducted proteomic expression analysis of COL1A1, COL1A2, COL3A1, and FN1 in GC samples compared to controls using the UALCAN database. The results revealed that the protein levels of these genes were significantly higher in GC samples compared to controls (Figure 5). The findings were consistent with the mRNA expression data, further validating the up-regulation of COL1A1, COL1A2, COL3A1, and FN1 in GC relative to control samples.

### Promoter methylation levels of COL1A1, COL1A2, COL3A1, and FN1

**Figure 6:**
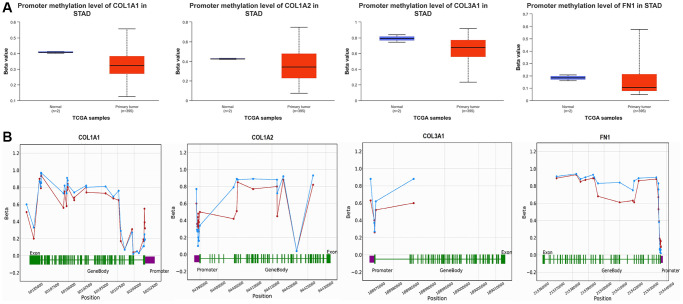
**Promoter methylation and survival analyses of COL1A1, COL1A2, COL3A1, and FN1.** (**A**) Promoter methylation analysis of COL1A1, COL1A2, COL3A1, and FN1 in gastric cancer (GC) and normal samples via UALCAN. (**B**) Promoter methylation analysis of COL1A1, COL1A2, COL3A1, and FN1 in GC and normal samples via OncoDB. *P*-value < 0.05.

In our study, we conducted promoter methylation analysis of COL1A1, COL1A2, COL3A1, and FN1 in GC samples compared to controls using the UALCAN and OncoDB databases. The results revealed that these genes exhibited hypomethylation in GC samples relative to controls (Figure 6). This finding suggests that the promoter regions of COL1A1, COL1A2, COL3A1, and FN1 undergo reduced methylation levels in GC, which could potentially contribute to their up-regulation of these genes in GC (Figure 6).

### Mutational and co-express gene analysis of COL1A1, COL1A2, COL3A1, and FN1

**Figure 7:**
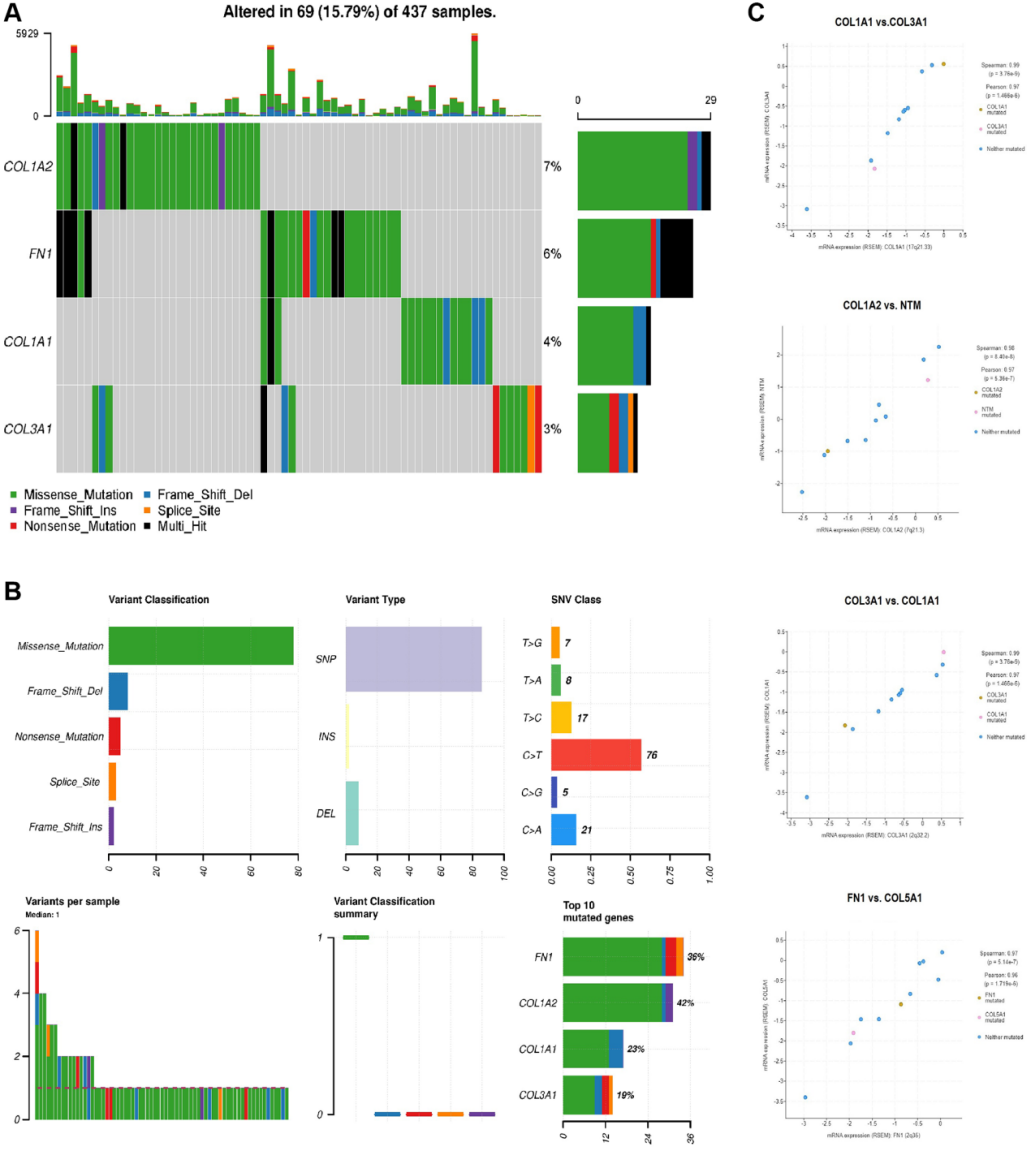
**Mutational and co-express gene analysis of COL1A1, COL1A2, COL3A1, and FN1.** (**A**) Detail of the mutational frequencies of COL1A1, COL1A2, COL3A1, and FN1 gens in gastric cancer (GC) samples. (**B**) Detailed summary of the mutations found in COL1A1, COL1A2, COL3A1, and FN1 genes across GC samples. (**C**) Significant co-expressed genes along with overexpressed COL1A1, COL1A2, COL3A1, and FN1 genes in GC samples. *P*-value < 0.05.

We conducted a mutational analysis of COL1A1, COL1A2, COL3A1, and FN1 in GC samples using the cBioPortal database. The results revealed that these genes were mutated in approximately 15.79% of the analyzed GC samples (Figure 7A). Among the mutated GC samples, missense mutations were particularly prevalent, with C>T substitution mutation being the most common type of mutation observed (Figure 7B). These findings suggest that these hub genes undergo genetic alterations in a subset of GC cases, and the prevalence of missense mutations, particularly C>T substitutions, underscores their potential significance in the molecular landscape of GC.

Additionally, in our study, we observed that COL1A1, COL1A2, COL3A1, and FN1, being the hub genes, exhibited co-expression patterns with other genes in GC samples. Notably, the co-expression analysis revealed that MTM and COL5A1 were among the genes showing significant co-expression with the hub genes (Figure 7C). This finding suggests that MTM and COL5A1 might be functionally linked to the hub genes and potentially involved in shared biological processes or pathways related to GC development and progression.

### Survival analysis and the construction of a prognostic model

**Figure 8:**
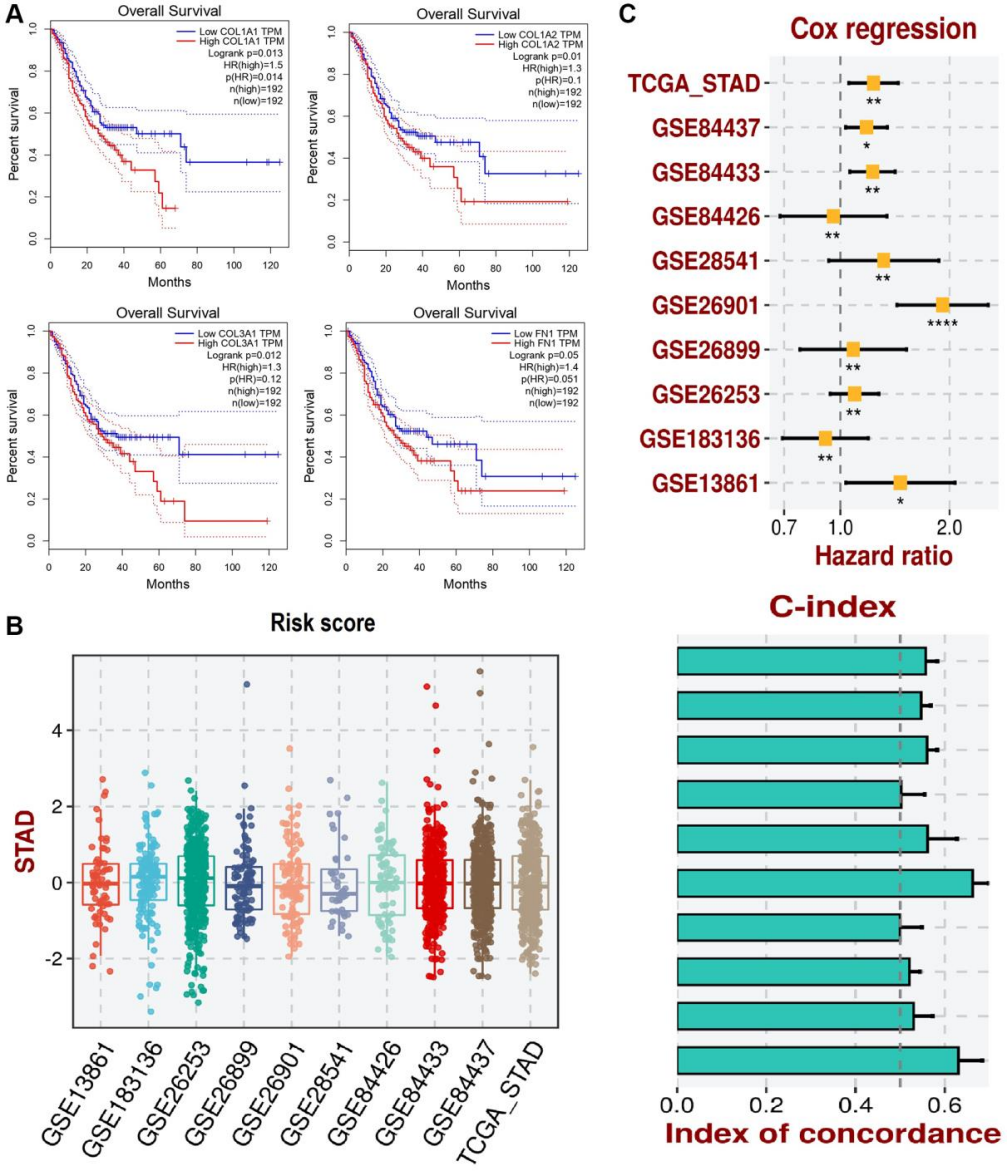
**This figure illustrates the survival analysis and development of a prognostic model using the gene expression data of COL1A1, COL1A2, COL3A1, and FN1.** (**A**) The survival analysis of these genes in gastric cancer (GC) patients is conducted via GEPIA. (**B**) Box plots representing the risk scores of patients in various GEO datasets and the TCGA_STAD dataset. (**C**) Cox regression analysis forest plot showing the hazard ratios of COL1A1, COL1A2, COL3A1, and FN gene expression levels for overall survival in STAD across different datasets and concordance index (C-index) bar plot for the predictive performance of the COL1A1, COL1A2, COL3A1, and FN gene expression models. The yellow squares represent the hazard ratio (HR) for each dataset with 95% confidence intervals. *P*-value < 0.05.

We conducted survival analysis of COL1A1, COL1A2, COL3A1, and FN1 in GC patients using the GEPIA database. The results revealed a significant association between higher expression levels of these genes and worse overall survival (OS) of the GC patients. This finding suggests that increased expression of COL1A1, COL1A2, COL3A1, and FN1 may be indicative of poorer prognosis in GC (Figure 8A).

To develop the prognostic model based on COL1A1, COL1A2, COL3A1, and FN1 genes, we utilized the TCGA_STAD dataset as the training dataset, and the GSE84437, GSE84433, GSE84426, GSE28541, GSE26901, GSE26899, GSE26253, GSE183136, and GSE13861 datasets served as validation datasets. The construction of our prognostic model involved a stepwise Cox regression approach, which integrated hazard ratio, c-index, and risk score parameters. By evaluating the predictive performance of our prognostic model using the c-index, we confirmed its effectiveness and robustness in assessing the prognosis of patients with GC (Figure 8B, 8C). The incorporation of multiple datasets for validation strengthens the reliability of our prognostic model and supports its potential clinical utility in predicting patient outcomes in GC.

### Enrichment and miRNA prediction analyses

**Figure 9:**
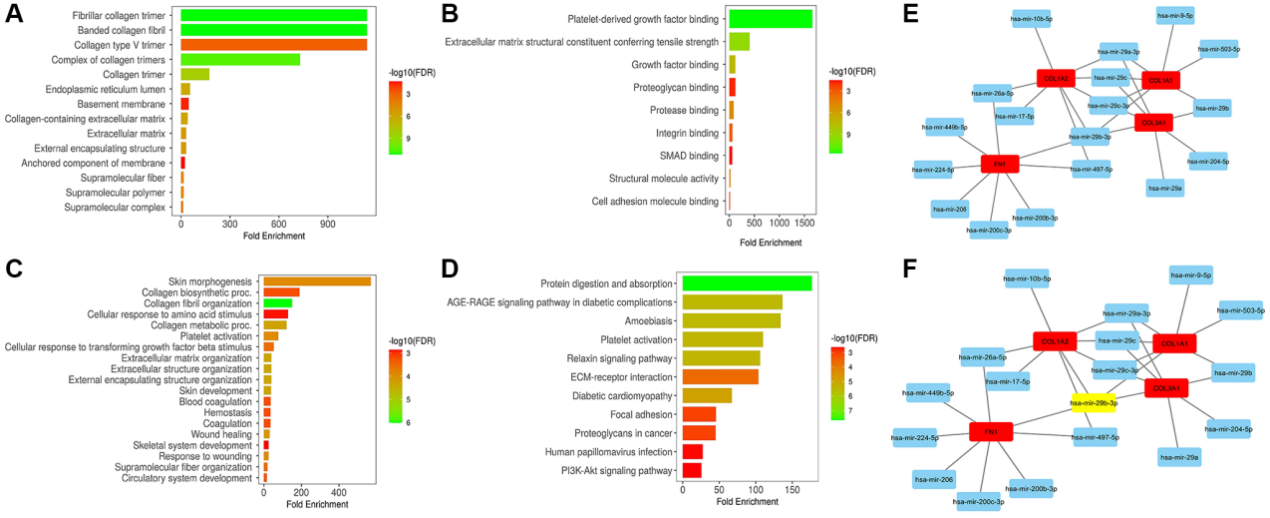
**This figure showcases the gene enrichment and miRNA prediction analyses of COL1A1, COL1A2, COL3A1, and FN1.** (**A**) Displays the associated cellular component (CC) terms. (**B**) Illustrates the associated molecular function (MF) terms. (**C**) Presents the associated biological process (BP) terms. (**D**) Shows the associated Kyoto Encyclopedia of Genes and Genomes (KEGG) terms. (**E**) Exhibits a protein-protein interaction (PPI) network of COL1A1, COL1A2, COL3A1, and FN1 along with their associated 40 miRNAs. (**F**) Demonstrates another PPI network of these genes and 40 miRNAs, highlighting the most significant miRNA (has-miR-29b-3p) in the network. *P*-value < 0.05.

In our study, we conducted enrichment analysis of COL1A1, COL1A2, COL3A1, and FN1 to gain insights into their functional roles. The analysis revealed that these hub genes are involved in a wide range of diverse GO terms and KEGG) pathways (Figure 9A–9D). These findings indicate that COL1A1, COL1A2, COL3A1, and FN1 may play crucial roles in various BP, CC, and MF.

Furthermore, in our study, we utilized miRDB and ENCORI to predict the regulatory miRNAs targeting COL1A1, COL1A2, COL3A1, and FN1. The analysis via both databases revealed a total of 40 miRNAs that potentially target these hub genes (Figure 9E). Remarkably, hsa-miR-29b-3p was found to target all hub genes simultaneously (Figure 9F). This observation suggests that hsa-miR-29b-3p may play a crucial role in the post-transcriptional regulation of COL1A1, COL1A2, COL3A1, and FN1, potentially modulating their expression levels.

### Validation of COL1A1, COL1A2, COL3A1, and FN1 gene expression in clinical GC samples via RT-qPCR

**Figure 10:**
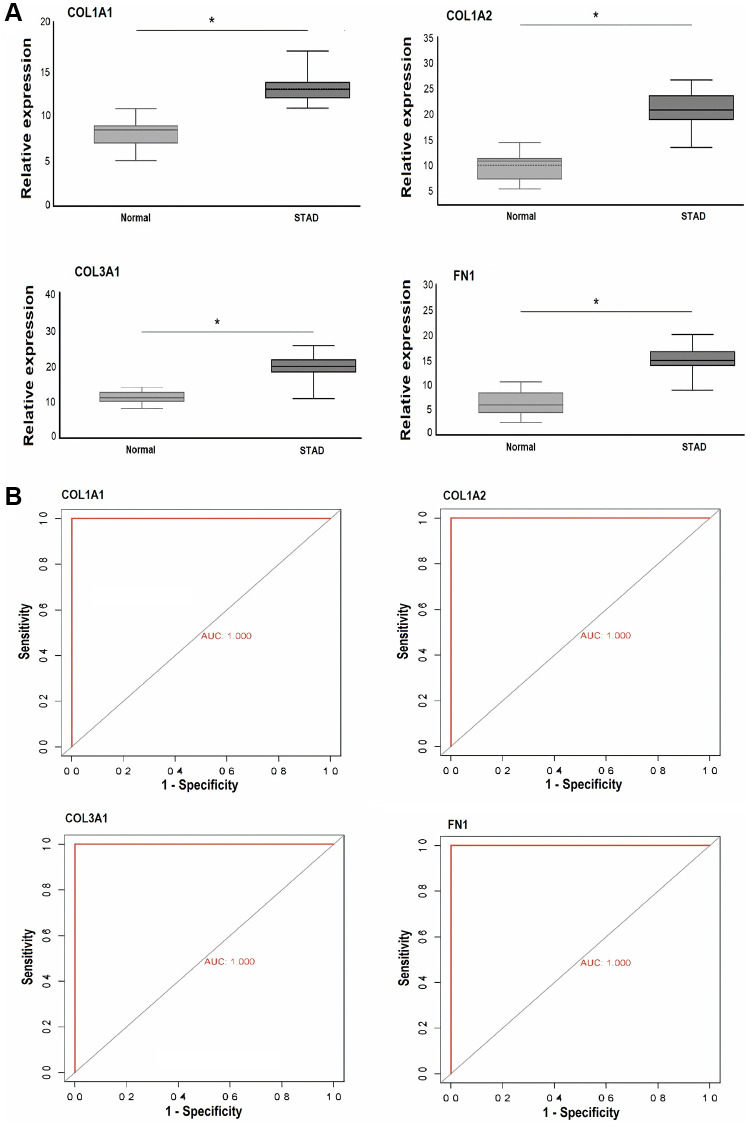
**This figure depicts the relative expression and receiver operating characteristic (ROC) curve analysis of COL1A1, COL1A2, COL3A1, and FN1 in Pakistani gastric cancer (GC) patients and normal controls.** (**A**) Presents the relative expression analysis of these genes in Pakistani GC patients and control samples via RT-qPCR. (**B**) Shows the ROC curves based on RT-qPCR expression of COL1A1, COL1A2, COL3A1, and FN1. A significance level of *P* < 0.05 was utilized as the selection criteria. *P*-value < 0.05.

To validate the results obtained from the GEO expression dataset, cDNA from both GC and control tissue samples was utilized for RT-qPCR analysis of COL1A1, COL1A2, COL3A1, and FN1. The results, as depicted in Figure 10A, demonstrated a significant increase in the expression levels of COL1A1, COL1A2, COL3A1, and FN1 in the GC sample group (*n* = 39) compared to the control group (*n* = 39, *p*-value < 0.05). Additionally, the ROC curves for COL1A1 (AUC: 1.0, *p*-value < 0.05), COL1A2 (AUC: 1.0, *p*-value < 0.05), COL3A1 (AUC: 1.0, *p*-value < 0.05), and FN1 (AUC: 1.0, *p*-value < 0.05) based on the expression levels exhibited significant diagnostic potential, sensitivity, and specificity (Figure 10B).

### Targeted bisulfite-seq analysis to analyze promoter methylation levels of COL1A1, COL1A2, COL3A1, and FN1 in clinical GC samples

**Figure 11:**
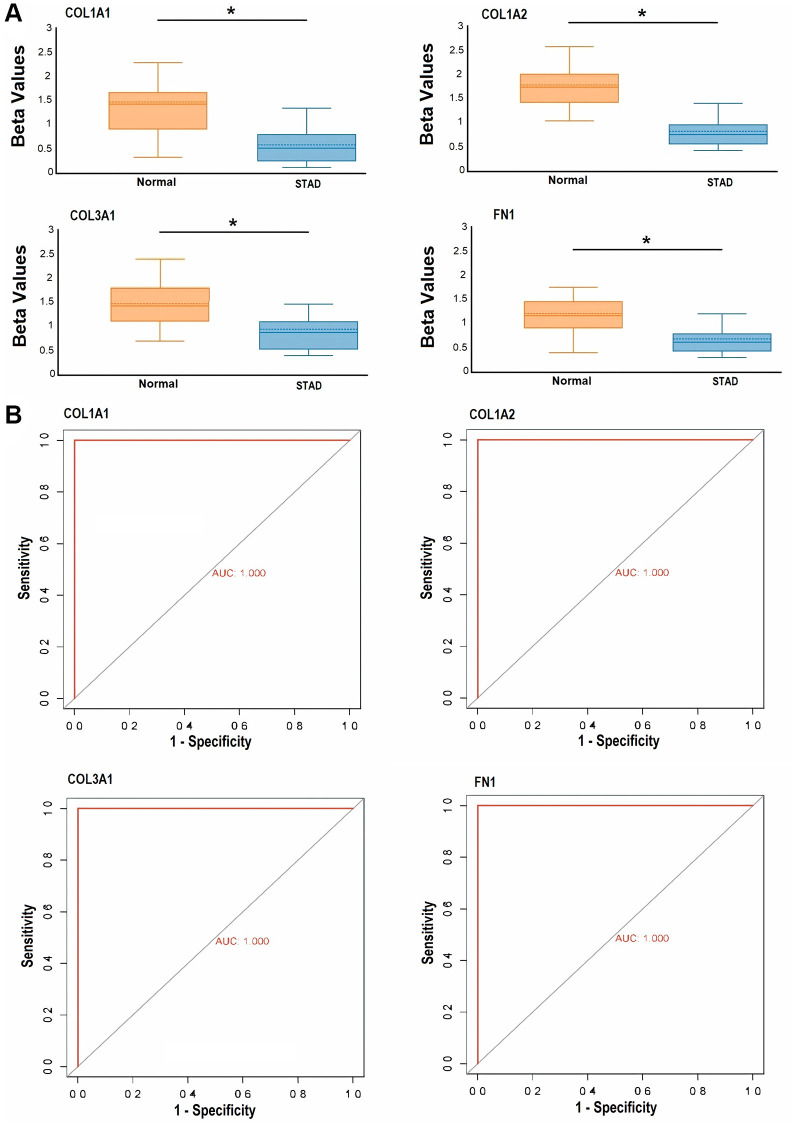
**Targeted bisulfite sequencing-based methylation level exploration and receiver operating characteristic (ROC) curve analysis of the hub genes, including COL1A1, COL1A2, COL3A1, and FN1 in Pakistani gastric cancer (GC) patients and normal controls.** (**A**) Beta value-based methylation analysis of COL1A1, COL1A2, COL3A1, and FN1 in Pakistani GC patients and control samples, and (**B**) targeted bisulfite sequencing-based ROC curves of the COL1A1, COL1A2, COL3A1, and FN1 methylation level. *P*-value < 0.05.

To assess the extent of promoter methylation in the hub genes COL1A1, COL1A2, COL3A1, and FN1 within clinical GC samples, we enrolled a total of 39 individuals diagnosed with GC, along with 39 healthy individuals from the Pakistani population. In both the GC and control groups, a high rate of bisulfite conversion (C to T) exceeding 99.1% was observed, and there were no notable differences in the read mapping rate between the two groups. Following stringent quality control measures, all 39 samples from the GC group and 39 samples from the control group were deemed suitable for subsequent analysis. Our analysis revealed a significant pattern of hypomethylation across all candidate genes (COL1A1, COL1A2, COL3A1, and FN1) in GC samples compared to the control group (Figure 11A). Furthermore, the ROC curves were generated for COL1A1 (AUC: 1.0, *p*-value < 0.05), COL1A2 (AUC: 1.0, *p*-value < 0.05), COL3A1 (AUC: 1.0, *p*-value < 0.05), and FN1 (AUC: 1.0, *p*-value < 0.05) based on their methylation levels (Figure 11B). These ROC curves demonstrated significant diagnostic potential with high AUC values of 1.0, indicating excellent discriminatory power. Additionally, the ROC curves exhibited remarkable sensitivity and specificity in distinguishing between GC and controls (Figure 11B).

### Drug prediction analysis

The management of GC often involves medical treatment as the primary approach. Therefore, the careful selection of suitable candidate drugs becomes essential. In this current investigation, we utilized the DrugBank database to explore potential therapeutic drugs for GC, focusing on the identified hub genes (COL1A1, COL1A2, COL3A1, and FN1) as potential targets for treatment. Notably, our investigation yielded two important drugs deemed suitable for the treatment of GC with respect to identified hub genes, namely Acetaminophen and Cytarabine (Table 1).

**Table 1:** DrugBank-based DEGs-associated drugs.

**Sr. no**	**Hub gene**	**Drug name**	**Effect**	**Reference**	**Group**
1	COL1A1	Acetaminophen	Decrease expression of COL1A1 mRNA	A20418	Approved
		Cytarabine		A20508	
2	COL1A2	Acetaminophen	Decrease expression of COL1A2 mRNA	A20418	Approved
		Cytarabine		A20508	
3	COL3A1	Acetaminophen	Decrease expression of COL3A1 mRNA	A20418	Approved
		Cytarabine		A20508	
4	FN1	Acetaminophen	Decrease expression of FN1 mRNA	A20418	Approved
		Cytarabine		A20508	

### Functional verification of the *in vitro* and *in vivo* roles of COL1A1, COL1A2, COL3A1, and FN1 in GC

**Figure 12:**
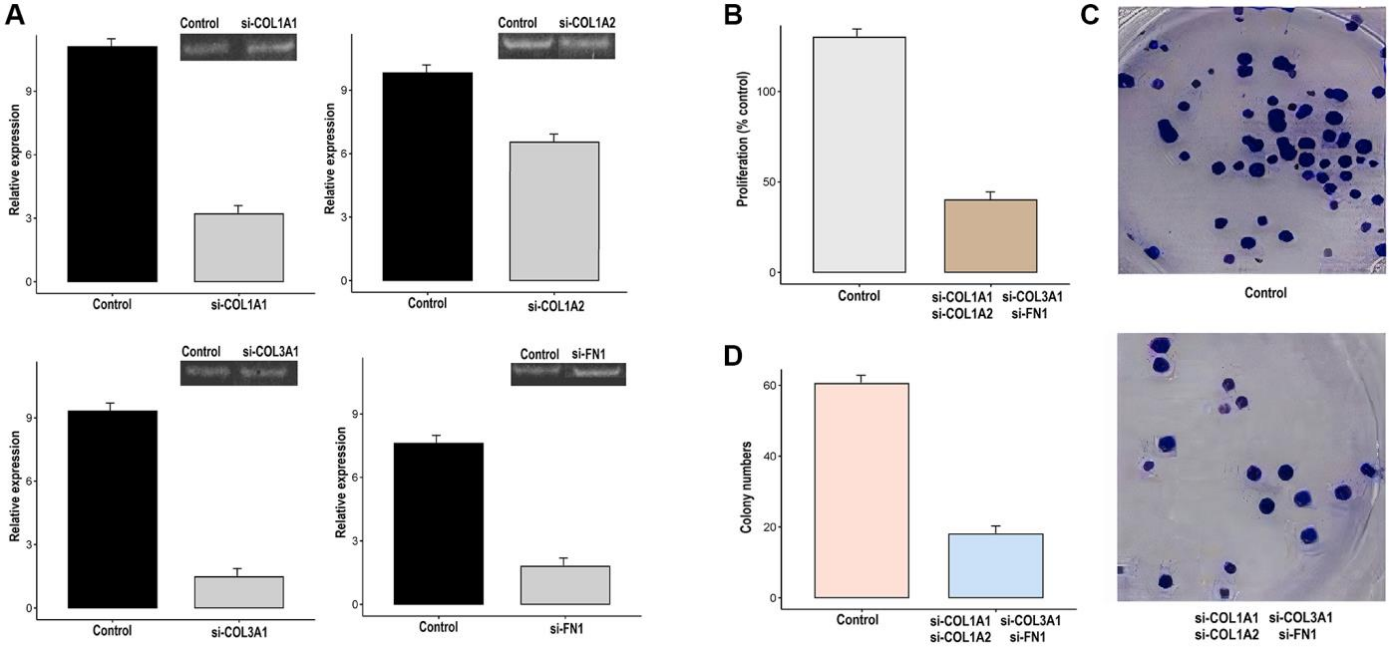
**Knockdown of COL1A1, COL1A2, COL3A1, and FN1 impairs the growth and metastatic potential of gastric cancer (GC) cells (AGS).** (**A**) The transfection efficiency of si-COL1A1, si-COL1A2, si-COL3A1, and si-FN1 was checked with the help of RT-qPCR, (**B**) AGS control and transfected cells were analyzed proliferation, (**C**, **D**) Colony formation.

The COL1A1, COL1A2, COL3A1, and FN1 genes work synergistically to regulate processes such as cell migration, invasion, and tissue remodeling. Therefore, the simultaneous silencing of COL1A1, COL1A2, COL3A1, and was carried out in AGS cells using siRNA to analyze their functional synergetic impact on the different parameters. The silencing efficiency was checked with the help of RT-qPCR. As shown in Figure 12A, reduced expression of COL1A1, COL1A2, COL3A1, and FN1 was observed in transfected AGS cell as compared to control AGS cells (Figure 12A). Further assessments, via CCK-8 assays and colony-forming assays, indicated that the knockdown of COL1A1, COL1A2, COL3A1, and FN1 led to a reduction in cellular proliferation when compared to the control AGS cells (Figure 12B–12D).

## DISCUSSION

In this study, we initially integrated three microarray expression profiles obtained from the GEO database, leading to the identification of 83 DEGs between GC and normal gastric tissues, with 41 up-regulated and 42 down-regulated genes. Subsequently, utilizing the degree and MCC methods, we designated COL1A1, COL1A2, COL3A1, and FN1 as hub genes, which exhibited significant up-regulation in GC. Furthermore, we validated the expression of these hub genes on additional GC datasets from TCGA and clinical samples collected from Pakistani GC patients. The expression validation analysis further confirmed the significant up-regulation of COL1A1, COL1A2, COL3A1, and FN1 in GC patients compared to controls.

COL1A1, encoding the alpha-1 chain of collagen type I, plays a critical role in the extracellular matrix (ECM) and is essential for maintaining tissue integrity and strength [33]. This protein is known to be involved in cell adhesion, migration, and proliferation, making it a key player in various biological processes [34]. Dysregulation of COL1A1 has been implicated in tumorigenesis and cancer progression in multiple malignancies. Research has demonstrated the significance of COL1A1 in different cancers. For instance, in breast cancer, up-regulated COL1A1 has been associated with tumor growth, invasion, and metastasis, promoting a pro-tumorigenic microenvironment [35]. Similarly, in pancreatic cancer, higher expression of COL1A1 has been found to enhance tumor cell proliferation and migration [36]. In hepatocellular carcinoma, overexpressed COL1A1 contributes to tumor progression and metastasis by modulating the tumor microenvironment [37]. In lung cancer, COL1A1 higher expression has been linked to tumor invasiveness and poor patient prognosis [38].

COL1A2, which codes for the alpha-2 chain of collagen type I, plays a critical role as a fundamental building block in the extracellular matrix (ECM), ensuring the integrity and strength of various tissues [39]. Like COL1A1, COL1A2 is involved in various cellular processes, including cell adhesion, migration, and proliferation, making it an important player in cancer biology [40]. Emerging research has shed light on the role of COL1A2 in different cancer types. For example, in breast cancer, up-regulation of COL1A2 has been associated with increased tumor invasiveness and metastasis [41]. In GC, COL1A2 has been identified as a potential biomarker for tumor progression and prognosis [42]. Moreover, in lung cancer, COL1A2 expression has been linked to tumor growth and metastasis [43]. In ovarian cancer, COL1A2 has been associated with tumor cell proliferation and migration [44]. In colorectal cancer, COL1A2 has been found to play a role in tumor invasion and metastasis [45]. Taken together, the evidence highlights the importance of COL1A2 in various cancers, with its dysregulation contributing to tumor aggressiveness and metastasis.

COL3A1, encoding the alpha-1 chain of collagen type III, is an essential component of the extracellular matrix (ECM) that provides structural support and elasticity to tissues [46]. Research has highlighted the role of COL3A1 in various cancer types. Such as, in colorectal cancer, elevated COL3A1 expression has been associated with tumor growth, progression, and metastasis, indicating its potential as a prognostic marker [47, 48]. Similarly, in ovarian cancer, overexpressed COL3A1 has been found to promote tumor cell migration and invasion, contributing to disease aggressiveness [49]. Moreover, in hepatocellular carcinoma, higher expression of COL3A1 has been implicated in tumor growth and angiogenesis, affecting patient prognosis [50]. These studies underscore the significance of COL3A1 in cancer biology and highlight its potential as a therapeutic target and diagnostic marker.

FN1 encodes for a significant glycoprotein responsible for cell adhesion, migration, and tissue remodeling, playing a crucial role in these processes [51]. FN1 interactions with various components of the extracellular matrix (ECM) are essential for facilitating cell-matrix interactions and maintaining tissue organization [52]. Studies have provided valuable insights into the diverse role of FN1 in different cancer types. In breast cancer, elevated FN1 expression has been linked to tumor invasiveness and metastasis, contributing to poor patient outcomes [53]. Similarly, in pancreatic cancer, FN1 has been associated with tumor progression and resistance to therapy, indicating its potential as a therapeutic target [54]. Moreover, in GC, FN1 overexpression has been correlated with tumor aggressiveness and lymph node metastasis, suggesting its significance as a prognostic biomarker [55].

During present study, we observed hypomethylation of COL1A1, COL1A2, COL3A1, and FN1 promoter regions in GC. Previous studies have also reported dysregulation of COL1A1, COL1A2, COL3A1, and FN1 promoter methylation in various cancers. Hypermethylation of the promoters has been linked to the down-regulation of these genes in breast, gastric, and colorectal cancers, contributing to tumor growth and invasion [56–59]. Conversely, hypomethylation of these genes has been observed in ovarian and lung cancers, leading to their overexpression and association with aggressive tumor phenotypes [60, 61].

Survival analysis of COL1A1, COL1A2, COL3A1, and FN1 in GC patients revealed the relevance of these genes with poor OS. Earlier studies have also reported the association of COL1A1, COL1A2, COL3A1, and FN1 expression with OS in different other cancer patients. For example, in breast cancer, high expression of COL1A1 has been associated with worse OS and distant metastasis [40]. In lung cancer, increased COL1A2 expression has been correlated with poorer OS and advanced tumor stage [62]. Similarly, up-regulation of COL3A1 and FN1 has been linked to unfavorable OS in stomach and ovarian cancers, respectively [63, 64].

Additionally, the results of this study emphasized that hsa-miR-29b-3p is a shared regulator of COL1A1, COL1A2, COL3A1, and FN1 expression. Dysregulation of this miRNA may play a role in the abnormal expression of COL1A1, COL1A2, COL3A1, and FN1, potentially contributing to the observed alterations in their expression levels. The hsa-miR-29b-3p has been implicated in tumorigenesis and cancer progression in different cancer types. For instance, in breast cancer, overexpressed hsa-miR-29c-3p has been shown to inhibit tumor cell migration and invasion by targeting specific genes involved in metastasis [65]. In GC, up-regulated hsa-miR-29c-3p has been reported to suppress tumor growth and induce apoptosis, indicating its tumor-suppressive function [66]. Conversely, in colorectal cancer, elevated hsa-miR-29c-3p has been found to promote cancer cell proliferation and invasiveness, suggesting an oncogenic role in this context [67].

The study presents valuable insights into GC through comprehensive analysis of gene expression profiles. Despite its strengths, including multi-database integration and identification of key hub genes, several limitations warrant consideration. The relatively small sample size and heterogeneity across datasets may limit result generalization. Additionally, biological variability and confounding factors were not fully addressed, potentially impacting result accuracy. Moreover, validation methods primarily focused on *in silico* and *in vitro* analyses, lacking extensive clinical validation. Lastly, the study’s single-omics approach overlooks other molecular layers crucial for a holistic understanding of GC. Addressing these limitations in future research could enhance the study’s clinical relevance and translational impact.

## CONCLUSION

This extensive investigation, combining experimental analyses and computational approaches, enabled the identification of DEGs linked to GC. Among these DEGs, four promising biomarkers (COL1A1, COL1A2, COL3A1, and FN1) were discovered, demonstrating potential diagnostic and prognostic implications in GC. Additionally, these genes hold promise as therapeutic targets for the treatment of GC, presenting new opportunities for targeted interventions.
